# Anaplastic lymphoma kinase-special immunity and immunotherapy

**DOI:** 10.3389/fimmu.2022.908894

**Published:** 2022-07-25

**Authors:** Ye Guo, Hanfei Guo, Yongfei Zhang, Jiuwei Cui

**Affiliations:** Cancer Center, The First Hospital of Jilin University, Changchun, China

**Keywords:** anaplastic lymphoma kinase, tumor microenvironment, immune evasion, immunotherapy, immune checkpoint inhibitors

## Abstract

Alterations in the anaplastic lymphoma kinase (*ALK*) gene play a key role in the development of various human tumors, and targeted therapy has transformed the treatment paradigm for these oncogene-driven tumors. However, primary or acquired resistance remains a challenge. *ALK* gene variants (such as gene rearrangements and mutations) also play a key role in the tumor immune microenvironment. Immunotherapy targeting the *ALK* gene has potential clinical applications. Here, we review the results of recent studies on the immunological relevance of ALK-altered tumors, which provides important insights into the development of tumor immunotherapies targeting this large class of tumors.

## Introduction

Over the past few decades, the anaplastic lymphoma kinase (*ALK*) gene has been widely known for its role in human tumorigenesis (1). Various rearrangements (fusions), mutations, amplification, and alternative splicing of the *ALK* gene have been found in anaplastic large cell lymphoma (ALCL), inflammatory myofibroblastoma (IMT), non-small cell lung cancer (NSCLC), and other human tumors (2–4) (**Table 1**). Currently, *ALK* gene variants are considered drug targets for these tumors. However, primary or acquired resistance to tyrosine kinase inhibitors is almost unavoidable (5). Although immunotherapy in recent years has provided new hope for patients with a variety of tumors with poor treatment efficacy, the response of these patients with *ALK* gene abnormalities to immunotherapy has not been clarified. A large retrospective study showed that patients with at least one oncogenic driver alteration (*RET, ROS1, EGFR*, or *ALK*) are less likely to benefit from immune checkpoint inhibitor (ICI) monotherapy (6). Until recently, several preclinical and clinical studies suggested that ALK rearrangement may be involved in innate and adaptive immunity through various pathways and is associated with T cell activation, cytokine release, and tumor immune escape (7). In addition, chimeric antigen receptor (CAR-T) therapies and tumor vaccines targeting ALK rearrangements are under development.

**Table 1:** Summary of ALK variants.

Variation type	Tumor (ALK positive rate)	Primary variation site (percentage of all ALK positive tumor)
Fusion	Anaplastic large cell lymphoma (ALCL) (60%)	NPM-ALK (80%), TPM3-ALK (12-18%)
	Non-small cell lung cancer (NSCLC) (3-7%)	EML4-ALK (80%)
	Inflammatory myofibroblastoma (IMT) (50%)	TPM3/4-ALK (95%)
	Diffuse large B-cell lymphoma (DLBCL) (rare)	CLTC-ALK
	Acute myelomonocytic leukemia (AML) (rare)	RANBP2-ALK
	Breast cancer (2.4%)	EML4-ALK
	Colorectal cancer (0.05-0.19%)	EML4-ALK, SPTBN1-ALK
	Renal cell carcinoma (<1%)	TPM3-ALK, VCL-ALK
	Thyroid carcinomas (1-3%)	STRN-ALK (50%), EML4-ALK (39%)
	Epithelioid fibrous histiocytoma (88%)	SQSTM1-ALK (52%), VCL-ALK (30%)
	Spitz tumors (10%)	DCTN1-ALK, TPM3-ALK (over 90%)
	Ovarian cancer (rare)	FN1-ALK, EML4-ALK
	Esophageal squamous cell carcinoma (ESCC) (rare)	TPM4-ALK
	Pancreatic cancer (rare)	EML4-ALK (over 50%)
Mutation	Neuroblastoma (15%)	F1174, F1245, R1275 (85%)
	Anaplastic thyroid cancer (ATC) (11%)	L1198F, G1201E
	ALK inhibitor-resistant NSCLC (30-50%)	L1196M
	ALK inhibitor-resistant ALCL	G1269A
	ALK inhibitor-resistant IMT	F1174L
Overexpression	Melanoma, Ovarian cancer, NSCLC, Breast cancer, Neuroblastoma, Astrocytoma, Glioblastoma, Ewing’s sarcoma, Colorectal cancer, Retinoblastoma, Rhabdomyosarcoma	

So far, ALK fusions have been found in more than 10 kinds of tumors (both hematopoietic neoplasms and solid tumors), and more than 100 fusion partners have been reported. In most cases, ALK fusions arise from the fusion of 3′ half of ALK, which retains its kinase catalytic domain, and the 5′ portion of a different gene that provides its promoter; The mutations of ALK are located in the kinase domain; ALK overexpression has been reported in various cancer types and cell lines, but its mechanism and its relationship with tumor drivers are still unclear.

Therefore, clarifying whether preferred targeted therapy, immunotherapy, or targeted combination immunotherapy is the optimal clinical treatment strategy for such patients is important. Hence, this topic will be the focus of future research in the field of ALK-altered tumor immunotherapy. This article reviews the progress on the knowledge of *ALK* gene variants in the field of immunotherapy to better understand the mechanism of ALK in the human immune response and may provide new treatment strategies for patients with *ALK* gene variants.

## Physiologic role of the *ALK* oncogene and its genetic aberrations in cancer

ALK, consisting of 1,620 amino acids, is a member of the insulin receptor tyrosine kinase (RTK) superfamily, and its gene is located on chromosome 2p23 (8). ALK plays an important role in the growth and development of the mammalian nervous system; however, its expression decreases significantly after birth and remains at a low level in adulthood (9). The tissue expression of ALK in human adults is restricted to the brain, with minimal expression in the lung, colon, small intestine, and testis, as indicated by the expression data of the human protein atlas and several immunohistochemical studies (10). When somatic variations occur, ALK is expressed in tissues that do not originally express ALK, and as such the cells are abnormally activated, resulting in uncontrolled cell proliferation and tumor formation (2, 11). Because ALK expression is restricted to the nervous system, a highly immune-privileged organ, the ALK protein is a potential antigen for the immune system. Similarly, tumor-specific ALK fusions or mutants may also be recognized as neoantigens in the body. Thus, ALK-altered cancer cells may potentially trigger antibody responses in patients. ALK is also involved in innate immunity against microbial pathogens (12, 13). Preclinical and clinical studies have shown that upregulation of immune-related molecules, such as programmed cell death ligand-1 (PD-L1), is commonly observed in ALK-altered tumors (14, 15).

## ALK variants affect the tumor microenvironment (TME)

**Figure 1:**
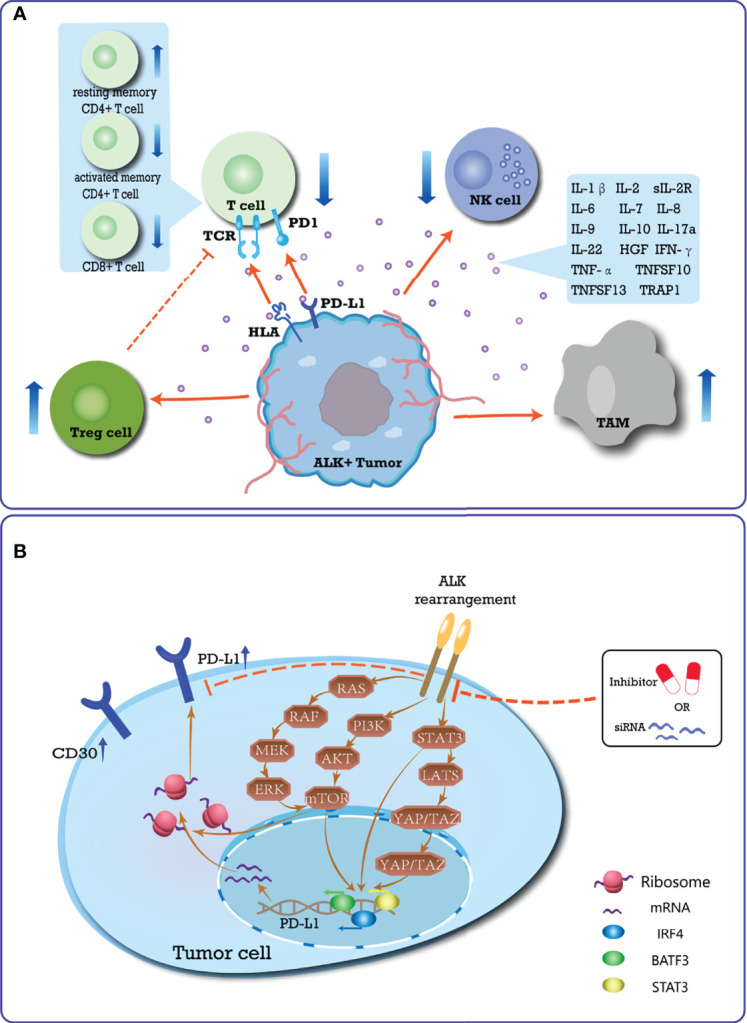
Summary of the immune-suppressive microenvironment induced by ALK-rearrangement. **(A)** Schematic diagram of the special immune TME of ALK-positive tumor. In ALK-positive tumors, CD30 is expressed continuously, and TCR signaling is inhibited. In the TME, the types of T cells changed, that is, the number of resting memory CD4+ T cells increased, while CD8+ T cells and activated memory CD4+ T cells were lacking. A variety of immunosuppressive cytokines are up-regulated, thereby inhibiting the killing ability of T cells and NK cells to tumor cells, and promoting the function of immunosuppressive cells. The special TME accumulates more Treg cells and TAM cells to promote immune evasion; **(B)** Mechanism of ALK rearrangement upregulating PD-L1 expression, which plays an essential role in mediating the process of PD-L1 expression. ALK-rearranged protein can activate STAT3, PI3K-AKT-mTOR, and MEK-ERK signaling networks, which upregulate PD-L1 expression through transcription factors acting on the promoter region of PD-L1 gene. Activated mTOR can also recruit PD-L1 transcripts to active polysomes at the post-transcriptional level. The JAK-STAT3-LATS-YAP/TAZ-PD-L1 signaling pathway has gradually been shown to play an important role in mediating ALK-induced upregulation of PD-L1 in multiple cancer cell lines. Conversely, blocking the activation of the ALK pathway inhibits the expression of PD-L1.

The mechanisms by which ALK-altered tumors lead to immune resistance may include affecting T cell immune responses, regulating cytokine secretion, activating immunosuppressive cells, and upregulating the expression of heterogeneous immune checkpoints (**Figure 1**).

### Effects of ALK variants on T cell response

In ALK-positive ALCL patients, CD30 is continuously expressed in tumor cells. Compared with CD30- tumors, CD30+ tumors are characterized by downregulation of molecules involved in T cell differentiation/activation (including CD28, CD52, and CD69) and T cell receptor (TCR) signaling (16). CD3 and TCR are negatively expressed in more than 75% of cases, and CD8 expression is rare in T cells (17). In addition, two immunogenic ALK epitopes (P280-89 and p375-86) were identified to elicit cytotoxic T cell (CTL) responses *in vitro*, *in vivo*, and in human peripheral blood lymphocytes (PBLs) (18). The anti-ALK CTL generated from the PBL of healthy donors induces an antigen-specific HLA-A2.1 restricted response, which can effectively kill endogenous ALK-expressing tumor targets. Subsequent studies using a mouse model of vaccination identified that, in healthy donors, CD8+ T cells mainly show a naive phenotype, whereas effector and memory CD8+ T cells are detected in ALK-positive ALCL patients (19). ALK-specific CD4+ T cells are detected in HLA-preselected ALCL patients using ALK-derived peptides (20). Recent studies have shown that the *in vitro* transduction of normal human CD4+ T lymphocytes by NPM-ALK leads to immortalization and malignant transformation (21). Moreover, tumor cells have the morphology and immunophenotype of primary anaplastic large cell lymphoma (21). In ALK+ NSCLC patients, Jin et al. (22) found that tumors are characterized by enriched resting memory CD4+ T cells (P<0.001), as well as a lack of CD8+ T cells (P<0.01), and activated memory CD4+ T cells (P=0.001).

### Relationship between ALK variants and cytokines

Various pro-inflammatory cytokines and their receptors are significantly upregulated in ALK-positive tumors, including IL-1β, IL-2, soluble IL-2 receptor (sIL-2R), IL-6, IL-7, IL-8, IL-9, IL-10, IL-17a, IL-22, interferon (IFN)-γ, TNF-α, TNFSF10, TNFSF13, hepatocyte growth factor (HGF), CD30, and TRAP1 (23–26). IL-9 and IL-22 activate oncogenic signaling *via* the JAK3-STAT3 pathway, and neutralizing antibodies against them may inhibit the survival and clonogenicity of ALK+ ALCL cells (27, 28). Furthermore, NPM-ALK promotes the expression of other immunosuppressive signals through the activation of STAT3, including IL-10 and transforming growth factor β (TGFβ) (29). Compared with ALK- ALCL, ALK+ ALCL patients are enriched for the expression of signatures of HIF1-α target genes, IL10-induced genes, and H-ras/K-ras induced genes (30).

### ALK variants activate immunosuppressive cells

Upregulation of IL6 and IL10 expression in ALK+ tumors reduce the antigen-presenting activity of dendritic cells in the TME and inhibits the function of T and NK cells (31, 32), resulting in ALK+ tumors responding to T cells and innate immunity negative effects. Upregulation of CSF1 and CCL18 expression in ALK+ tumors increase M2 tumor−associated macrophages (TAMs) in the TME that contribute to immune evasion (33–35). Previous studies have identified that ALK-mediated activation of TMEM173 (transmembrane protein 173, also known as STING) in macrophages and monocytes is related to the pathogenesis of sepsis caused by infection, and has the potential to activate macrophages and monocytes (12, 36). Recently, Jan et al. compared the immune gene expression profiles and the levels of specific immune cell populations in ALK+ and ALK- lung adenocarcinoma patients. In ALK+ tumors, the proportion of regulatory T cells was significantly increased (P < 0.0005) (35). Further analysis revealed that ALK+ tumors recruit CXCR4+ Tregs by upregulating CXCL12 and CCL22 (35, 37, 38). These studies all showed that ALK variants can activate immune suppressive cells, presenting a challenge to immune-related treatment of patients with ALK+ tumors.

### ALK variants affect the expression of immunosuppressive molecules

Mutant ALK upregulates the expression of PD-L1, which may potentially confer an immunosuppressive TME, contributing to tolerance and immune evasion in cancer (39, 40). Marzec et al. (29) showed that, in an ALK+ ALCL cell model, NPM-ALK activates the transcription of STAT3 on the PD-L1 promoter. Using CRISPR/Cas9 library screening, Zhang et al. determined that PD-L1 induction is dependent on the NPM-ALK oncoprotein activation of STAT3, as well as a signalosome containing GRB2/SOS1, which activates the MEK-ERK and PI3K-AKT signaling pathways. These signaling networks ultimately induce PD-L1 expression through the action of the transcription factors IRF4 and BATF3 on the enhancer region of the *PD-L1* gene (41). A recent clinical study conducted by the MD Anderson Cancer Center of 95 patients with ALCL showed that the positive rate of PD-L1 in ALK+ ALCL patients is higher than that in ALK- cases (76% and 42%, respectively) (42). The same phenomenon was observed in patients with ALK+ NSCLC. Both *in vitro* and *in vivo* experiments have shown that the expression level of PD-L1 is positively associated with the presence of EML4-ALK in NSCLC specimens (43–46). EML4-ALK modulates PD-L1 expression *via* common downstream signaling pathways mediated by PI3K-AKT-mTOR, MEK-ERK, and STAT3 (44, 47, 48). Activated mTOR recruits PD-L1 transcripts to active polysomes at the post-transcriptional level, thereby increasing the level of PD-L1 protein without significantly increasing the mRNA levels (49, 50). STAT3 increases PD-L1 transcription by directly binding to the promoter region of the *CD274* gene (located at the 9p24.1 locus) (47). Recently, Nouri et al. (51) identified, through the kinome-wide screen of Hippo pathway regulators, that YAP/TAZ are critical in mediating ALK-induced upregulation of PD-L1 in multiple cancer cell lines. Moreover, ALK may cause enhanced immune evasion and tumorigenesis through the JAK-STAT3-LATS-YAP/TAZ-PD-L1 signaling pathway. Importantly, ALK inhibitors and ALK siRNAs effectively inhibit ALK fusion-induced PD-L1 expression in NSCLC cell models. These results confirmed the effect of ALK on PDL1 expression in NSCLC (44, 52).

## Current landscape of immunotherapy of ALK-altered tumors

Various preclinical and clinical efforts are underway to identify mechanisms related to the interaction of the *ALK* gene with the tumor immune microenvironment. ICIs targeting programmed cell death ligand-1 (PD-1), PD-L1, and cytotoxic T lymphocyte-associated antigen 4 (CTLA-4) are currently the most advanced immunotherapies and have transformed the treatment paradigm for a variety of tumors, including lung cancer. However, there is no firm conclusion regarding the therapeutic effect of ICIs in patients with ALK-altered tumors. Research on tumor vaccines and chimeric antigen receptor T-Cell (CAR-T cell) therapy targeting ALK are also underway (**Table 2**).

**Table 2:** Summary of ongoing trials with immunotherapy in ALK+ tumors (source: www.clinicaltrials.gov, last accessed: 30 Mar 2022).

Clinical Trial Identifier	Phase	Tumor	Study Title	Setting	N	Experimental Arm	Control Arm(s)	Primary Outcome (s)
NCT04042558	II	NSCLC	A Study Evaluating Platinum-Pemetrexed-Atezolizumab (+/-Bevacizumab) for Patients With Stage IIIB/IV Non-squamous Non-small Cell Lung Cancer With EGFR Mutations, ALK Rearrangement or ROS1 Fusion Progressing After Targeted Therapies (GFPC 06-2018)	PD-L1/anti-angiogenesis	149	Carboplatin + Pemetrexed + Atezolizumab + Bevacizumab	Carboplatin + Pemetrexed + Atezolizumab	ORR
NCT03991403	III	NSCLC	Study of Atezolizumab in Combination With Carboplatin + Paclitaxel +Bevacizumab vs With Pemetrexed + Cisplatin or Carboplatin With Stage IV NON-SQUAMOUS NON-SMALL CELL LUNG CANCER With EGFR(+) or ALK(+)	PD-L1/anti-angiogenesis	228	Atezolizumab+Carboplatin + Paclitaxel +Bevacizumab	Pemetrexed+Carboplatin/cisplatin	PFS
NCT02393625	I	NSCLC	A Multi-center, Open-label Study to Assess the Safety and Efficacy of Combination Ceritinib (LDK378) and Nivolumab in Adult Patients With Anaplastic Lymphoma Kinase (ALK)-Positive Non-small Cell Lung Cancer (NSCLC)	PD-1	57	Ceritinib+Nivolumab		MTD and/or Recommended Dose for Expansion; ORR
NCT04425135	II	non-squamous NSCLC	Phase II Single-arm Clinical Study of Camrelizumab Combined With Apatinib Mesylate and Standard Chemotherapy (Pemetrixed +Carboplatin) in Patients With Tyrosine Kinase Inhibitor Failure in ALK-positive Advanced NSCLC	PD-1/anti-angiogenesis	59	Camrelizumab +apatinib mesylate+Pemetrixed + Carboplatin		ORR
NCT03703050	II	ALCL	Phase II Trial of Nivolumab for Pediatric and Adult Relapsing/Refractory ALK+ Anaplastic Large Cell Lymphoma, for Evaluation of Response in Patients With Progressive Disease (Cohort 1) or as Consolidative Immunotherapy in Patients in Complete Remission After Relapse (Cohort 2)	PD-1	38	Nivolumab		Best objective response rate; PFS
NCT02462538	I/II	ALCL	A “Window of Opportunity” Trial With Brentuximab Vedotin and Imatinib in Patients With Relapsed or Refractory ALK+ Anaplastic Large Cell Lymphoma or Patients Ineligible for Chemotherapy	CD30	10	Brentuximab vedotin + Imatinib		AEs
NCT02799095	I/II	Advanced Solid Tumors*	A Phase 1/2 Study of ALKS 4230 Administered Intravenously as Monotherapy and in Combination With Pembrolizumab in Subjects With Advanced Solid Tumors - ARTISTRY-1	IL-2	347	ALKS 4230 + pembrolizumab	ALKS 4230	DLT; AEs; ORR
NCT03861793	I/II	Advanced Solid Tumors*	A Phase 1/2 Study of ALKS 4230 Administered Subcutaneously as Monotherapy and in Combination With Pembrolizumab in Subjects With Advanced Solid Tumors - ARTISTRY-2 (001)	IL-2	185	ALKS 4230+Pembrolizumab	ALKS 4230	AEs; ORR

ORR, Objective response rate; PFS, Progression-free survival; AEs, Adverse events; MTD, Maximum tolerated dose; *All eligible patients can be included in the group, no genetic requirements.

### Immune checkpoint inhibitors (ICIs)

In recent years, ICIs have shown remarkable therapeutic effects in various tumors. Moreover, as mentioned above, ALK variants induce the upregulation of PD-L1 expression in ALK-positive tumors. Based on these findings and *in vitro* drug trials, some scholars have speculated that anti-PD-1/PD-L1 therapy may be a promising option for NSCLC patients with upregulated PD-L1 carrying the EML4-ALK fusion gene (53). However, whether the high expression of PD-L1 affects the prognosis of ALK+ patients remains inconclusive, and further research is needed (42, 54).

#### ICI monotherapy

Data from prior randomized studies indicate that immunotherapies are less effective in patients with ALK+ tumors than in those with wild-type tumors, regardless of PD-L1 expression level (55, 56). In a global “real world” study, Mazieres et al. (6) retrospectively analyzed ALK+ NSCLC patients from 10 countries and found that the objective response rate is 0% using ICI monotherapy. The proportion of ALK+ patients who experienced rapid progression within 2 months was 45.5%, which was much higher than that of patients with the wild-type gene. More recently, a multicenter retrospective study showed limited activity in patients with stage III unresectable NSCLC with driver genomic alterations treated with durvalumab (PD-L1 inhibitor) after chemoradiotherapy, especially in the ALK rearrangement subgroup. The median progression-free survival (PFS) was not reached (11.3-NR) in the KRAS-mutation vs. 8.1 month in the EGFR-mutation vs. 7.8 month in the BRAF-mutation/ALK rearrangement (P = 0.02) (57). Therefore, current research on ALK-positive patients has mainly focused on ALK inhibitor resistance (58). For patients with NSCLC, the ATLANTIC trial established an independent cohort of EGFR+/ALK+ patients to evaluate durvalumab as a third line or later treatment. The proportion of patients who achieved a response was generally lower in the cohort of patients with EGFR+/ALK+ NSCLC than in those with EGFR−/ALK− NSCLC. Nevertheless, the proportion of EGFR+/ALK+ patients with at least 25% of tumor cells expressing PD-L1 who achieved an objective response was not substantially lower than that in EGFR−/ALK− patients (12.2% vs 16.4%) (59). Recently, there was a report of a case of a 48-year-old man with ALK+ NSCLC who displayed a complete response for 16 months to nivolumab (PD-1 inhibitor) therapy in a third line setting after ceritinib (second-generation ALK inhibitor) and platin-based chemotherapy (60). Another case report showed that patients with ALK+ ALCL (PD-L1 positive) who were refractory to chemotherapy and ALK inhibitors demonstrated prolonged responses to nivolumab (61, 62). Further clinical trials are needed to verify the effectiveness of ICIs in patients with ALK+ ALCL.

Some studies have analyzed the reasons for the poor effects of ICIs. A majority of ALK-positive NSCLCs lack concurrent PD-L1 expression and high levels of CD8+ tumor infiltrating lymphocytes (TILs) (63). The combined analyses of PD-L1 and CD8+ TILs show a remarkably higher proportion of PD-L1-/TIL- tumors and a lower proportion of PD-L1+/TIL+ tumors in ALK+ groups than in wild-type patients (P = 0.001), suggesting an uninflamed phenotype with immunological ignorance (22). Although a significant number of PD-1 positive CD8+ T cells were found in the ALK-positive tumor bed in early lung adenocarcinoma (64), these PD-1 expressing CD8+ T cells were functionally impaired (65) and did not express interferon-γ mRNA, which could upregulate PD-L1 expression in tumor cells (66, 67). These results indicate that the ALK-positive TME suppresses the immune function of CD8+ TILs through a PD-1/PD-L1 independent mechanism, which might lead to the inability of ALK-positive tumors to respond to PD-1/PD-L1-based immunotherapy (64). Tumor mutational burden (TMB) is an effective marker for predicting the efficacy of ICI treatment. The median TMB of ALK-positive tumor samples is only 2.29 mutations/Mb (ranging from 0.76 to 16.79 mutations/Mb) (68). The TMB (in mutations/Mb) of NSCLC patients with alteration in ALK is significantly lower than in those without (2.1 vs 7.0 mutations/Mb; P < 0.001) (69). These results suggest that the limited benefits of ICI monotherapy are attributable to the low levels of functional CD8+ TILs and TMB.

#### ICIs combined with ALK tyrosine kinase inhibitors (ALK-TKIs)

A preclinical study showed that *in vitro* application of ceritinib combined with a PD-L1 inhibitor in the treatment of ALK-rearranged NSCLC promotes lymphocyte proliferation and activation, inhibits PD-L1 expression, and enhances lymphocyte cytotoxicity and cell death. In the *in vivo* xenograft model, tumor volumes treated with a combination of ceritinib and a PD-L1 inhibitor (91.9%) are significantly smaller than those treated with ceritinib (84.9%) or PD-L1 (20.0%) alone (70). Some clinical trials have explored the use of ICIs in combination with ALK inhibitors (71, 72). The primary study was a phase 1/2 study (CheckMate 370) on the safety and tolerability of nivolumab plus crizotinib (first-generation ALK inhibitor) as a first-line treatment for patients with advanced ALK+ NSCLC. The high proportion (38%) of severe hepatotoxicity caused the trial to close prematurely and fail (73). Another phase Ib study evaluated the safety and preliminary antitumor activity of crizotinib plus pembrolizumab (PD-1 inhibitor) as a first-line therapy in patients with ALK+ NSCLC. Although this combination showed antitumor activity, the incidence of dose-limiting toxicities is high, especially with a higher frequency of severe transaminase level increase. Because the study was terminated early, the recommended phase II dose could not be determined (74). Therefore, for a well-designed trial, selecting a suitable combination of partner and treatment population is extremely important. Felip et al. (75) presented the results of a phase Ib trial examining ceritinib plus nivolumab in previously treated or treatment-naive ALK+ NSCLC. This combination appears to elicit activity, and high PD-L1 expression may be enriched in patients more likely to respond. Based on more toxicity findings, especially rash, a protocol amendment to switch to sequential treatment is being investigated in which ceritinib is administered as monotherapy for two cycles before combining it with nivolumab. Two additional phase Ib studies presented at ASCO meeting show promising efficacy and acceptable safety profile of this sequential therapy. In previously treated ALK+ NSCLC, the combination of avelumab (anti-PD-L1) and lorlatinib (third-generation ALK inhibitor) showed no dose-limiting toxicity (76). In treatment-naive ALK+ NSCLC, alectinib (second-generation ALK inhibitor) should be administered 1 week prior to combination with atezolizumab (PD-L1 inhibitor). The objective response rate was 81% (95% CI 58.1–94.6), with a median PFS of 21.7 months and a median DOR of 20.3 months (77). In addition, Chalmers et al. presented a phase I trial of a combination of ipilimumab (a CTLA-4 inhibitor) and crizotinib in ALK+ NSCLC. The median PFS and overall survival (OS) were prolonged, but owing to the small number of enrolled cases (three cases), continued observation was necessary (78). Although a particularly large advantage in ORR was not observed in most combination therapies, given the long-term benefits of ICIs treatment, it remains to be seen whether PFS and OS outcomes can be prolonged in the future.

#### ICIs combined with anti-angiogenesis therapy

In the IMpower130 study, for ALK inhibitor-pretreated patients with ALK-sensitizing alterations, atezolizumab plus chemotherapy did not show improved overall survival versus chemotherapy alone (79). However, data from the IMpower150 study showed that the addition of atezolizumab to bevacizumab (angiogenesis inhibitor) plus chemotherapy resulted in significant improvements in PFS and OS (80). In IMpower150, the median PFS for patients with EGFR+/ALK+ status in the atezolizumab plus bevacizumab and chemotherapy (ABCP) group was 9.7 months compared with the PFS of 6.1 months in the bevacizumab plus chemotherapy (BCP) group (HR 0.59, 95% CI, 0.37–0.94). OS data were immature (not reached vs. 17.5 months; HR, 0.54; 95% 0.29–1.03). The 6- and 12-month PFS rates in the ABCP group were 65% and 37%, respectively, compared to 53% and 21% in the BCP group (80, 81). Therefore, after ALK inhibitor resistance, ABCP may be the first choice for patients with ALK+ NSCLC who are still capable of tolerating intensive therapy. The combination of ICIs and anti-vascular endothelial growth factor (VEGF) agents has significantly improved clinical outcomes in a variety of tumors compared with standard treatments (82). Multiple studies have further analyzed the synergistic mechanism between angiogenic factors such as VEGF and PD-(L)1 inhibitors, which is attributed to VEGF-mediated immunosuppression in the TME (83, 84). In addition to inducing vascular abnormalities, angiogenic factors also suppress antigen presentation and immune effector cells or augment the immunosuppressive activity of regulatory T cells, myeloid-derived suppressor cells, and tumor-associated macrophages (85–88). In the PI3K/AKT/mTOR pathway, ALK signaling promotes VEGF expression in tumors, which might enhance the sensitivity of ALK+ patients to bevacizumab (89). In ALK+ patients, CD8+ T cell tumor infiltration decreases (84) and regulatory T cells increase (90) after ALK inhibitor treatment, which induces a lower response rate to ICIs. In several clinical biomarker studies, the combination of bevacizumab and atezolizumab has been proven to overcome ICIs resistance by reversing VEGF-mediated immunosuppression and promoting CD8+ TIL in tumors (91–93). There are also reports that bevacizumab combined with targeted therapy can overcome ALK inhibitor resistance (94, 95). A recent study showed that VEGFR2 inhibition, a promising treatment strategy for oncogene-driven NSCLC, not only inhibits tumor angiogenesis but also exerts direct antiproliferative effects on cancer cells (96). In summary, it can be inferred that ICIs combined with anti-angiogenesis may be a promising treatment method.

### ALK vaccine

Owing to the characteristics of ALK expression in the body, it has long been considered a potential tumor-associated antigen (TAA) (97). There are immunogenic regions located in the ALK kinase domain that can trigger specific T cell responses restricted by HLA alleles (98, 99). These findings provide a basis for peptide vaccine immunotherapy for ALK-driven tumors.

Using an ALK+ ALCL mouse model, Chiarle et al. showed that DNA vaccines with plasmids encoding a part of the ALK cytoplasmic domain elicit ALK-specific interferon-gamma responses and CD8+ T cell-mediated cytotoxicity. The combination of chemotherapy and ALK DNA vaccination significantly enhances the survival of mice challenged with ALK+ lymphomas (100). In mouse models of ALK+ NSCLC, this ALK DNA vaccine induced strong systemic and intratumoral immune responses, significantly reducing tumor growth and extending the survival of treated mice. The combination of this vaccine and ALK TKI is also effective and significantly delayed tumor relapse after TKI treatment. In addition, immunotherapies, such as anti-PD-1/PD-L1 or anti-CTLA, can be used to enhance the benefits of ALK TKI and ALK vaccine combination therapy (101). Another ALK vaccine is based on ALK-overlapping peptides in splenocytes from ALK-vaccinated mice. The vaccine significantly delayed the progression of primary lung tumors in EML4-ALK transgenic mice (102). One of the technologies under study is the use of stabilized multilamellar lipid vesicles with cross-linked lipid bilayers containing an antigenic ALK variant. They can deliver antigens alone in the presence of adjuvants to form an efficient vaccine for ALK-positive glioblastomas (103). Recently, an *in vitro* test applied a novel anti-epidermal growth factor vaccine (anti-EGF VacAbs) in ALK+ NSCLC cell lines. The anti-EGF VacAbs target the B-cells to generate antibodies that neutralize circulating EGF, thus preventing its binding to EGFR. They potentiate the antitumor effects of ALK-TKIs, significantly enhancing the blockade of downstream oncogenic activation pathways, and delaying the emergence of resistance (104). These experimental results provide a powerful strategy for the treatment of ALK-driven tumors. With the continuous progress in its research, ALK vaccines will soon enter clinical trials.

### CAR-T cells & TCR-T cells

T cells engineered to express chimeric antigen receptors (CARs) have demonstrated significant activity against many tumors, and CAR-T cells have recently joined a rapidly growing repertoire of immunotherapeutics. Because ALK fusion protein is mainly expressed inside the cell, CAR-T therapy targeting ALK is currently mainly tested in neuroblastoma. It has been found that T cells expressing a CAR incorporating the single-chain variable fragment against the ALK extracellular domain lyse ALK-positive neuroblastoma cell lines. However, CAR functionality is regulated by target antigen and CAR density, and low expression of either contributes to the limited anti-tumor efficacy of ALK CAR-T (105, 106). More specific immunotherapies targeting ALCL surface markers include anti-CD30 CAR-T cells. CD30-specific CAR-T cells have been tested in mouse models and clinical trials have been initiated (107). In one case report, a patient with relapsed ALK+ ALCL achieved remission after CD30-specific CAR-T cell treatment (108). Another trial under investigation is the induction of an immunologic response in a tumor patient using mature dendritic cells transfected with a nucleic acid composition encoding NPM-ALK as a tumor antigen and loaded with a corresponding tumor antigen composition (103).

With the revolutionary breakthroughs in the field of TCR therapy in recent years, an increasing number of ALK epitopes/peptides may become suitable targets for directed immunotherapy (109, 110). An ongoing study is screening for autologous or allogeneic T cell receptor-transgenic T cells to test against ALK+/- patient-derived and cancer cell lines using *in vitro* and *in vivo* models to assess the potential utility of cytotoxic TCR-directed immunotherapies (111).

## Conclusion and prospects

In summary, ALK variants play an important role in a variety of tumors, including both hematological and solid tumors. The development and application of ALK inhibitors have made outstanding contributions to the treatment of ALK+ tumor patients, and it is still the main choice for first-line treatment (112). However, to date, resistance to ALK inhibitors has proven unavoidable in all cases (113). For TKIs resistant patients, the exploration of immunotherapy is currently a promising treatment direction. According to the special immunosuppressive microenvironment of ALK+ tumors, there are still huge challenges in the development and application of immunotherapeutic interventions. Based on the results of current clinical studies, ICIs monotherapy is not the preferred treatment option for TKI-resistant patients. We urgently need to explore better combined treatment options to change tumor immunosuppression to control tumors (114), such as immunotherapy combined with targeted therapy or anti-angiogenesis therapy. Nevertheless, there are still many obstacles in the process of exploration, including the understanding of the specific effects of ALK on the immune microenvironment and development of novel immunotherapy methods. Numerous studies are exploring new treatments and ways to optimize the application of immunotherapy, which may lead to greater survival benefits for the patients (**Table 2**).

## Author contributions

YG carried out the primary literature search, drafted and revised the manuscript. HG and YZ helped modify the manuscript and participated in discussions. JC conceived and approved the final manuscript. All authors contributed to the article and approved the submitted version.

## Funding

This work was supported by the National Natural Science Foundation of China (No. 81874052) and Jilin Scientific and Technological Development Program (CN) (No. 20190303146SF).

## Conflict of interest

The authors declare that the research was conducted in the absence of any commercial or financial relationships that could be construed as a potential conflict of interest.

## Publisher’s note

All claims expressed in this article are solely those of the authors and do not necessarily represent those of their affiliated organizations, or those of the publisher, the editors and the reviewers. Any product that may be evaluated in this article, or claim that may be made by its manufacturer, is not guaranteed or endorsed by the publisher.
